# Psychotherapy services: treatment gaps and unmet needs

**DOI:** 10.1192/j.eurpsy.2026.10291

**Published:** 2026-07-31

**Authors:** F. H. Kraxner

**Affiliations:** Forensic psychiatry, Psychiatric Services of Kanton Aargau (PDAG), Brugg/Windisch, Switzerland

## Abstract

**Abstract:**

Despite strong evidence for its effectiveness, psychotherapy remains insufficiently accessible across many European health systems, resulting in substantial treatment gaps and persistent unmet mental health needs. This presentation examines the current landscape of psychotherapy service provision in Europe, with a particular focus on the mismatch between population-level mental health needs and available psychotherapeutic care.

Drawing on comparative data and system-level observations, the talk will outline key structural and organizational factors contributing to treatment gaps, including workforce shortages, uneven geographic distribution of services, long waiting times, limited public reimbursement, and fragmentation between primary care, specialist services, and community-based care. Special attention will be given to vulnerable populations, for whom barriers to access further exacerbate existing health inequalities.

The presentation will also explore the clinical, public health, and societal consequences of unmet psychotherapeutic needs, including symptom chronicity, increased use of emergency and inpatient services, reduced functional outcomes, and broader economic costs. By situating these findings within a public mental health framework, the talk highlights how insufficient access to psychotherapy undermines both individual recovery and system sustainability.

In conclusion, the presentation emphasizes the need for coordinated policy, workforce development, and service-level strategies to reduce treatment gaps and improve equitable access to psychotherapy across Europe, contributing to higher-quality mental health care and expanded horizons for prevention and early intervention.

**Disclosure of Interest:**

None Declared

